# Fixed-Dose Combination of NSAIDs and Spasmolytic Agents in the Treatment of Different Types of Pain—A Practical Review

**DOI:** 10.3390/jcm10143118

**Published:** 2021-07-15

**Authors:** Magdalena Janczura, Małgorzata Kobus-Moryson, Szymon Sip, Marcin Żarowski, Agnieszka Wareńczak, Judyta Cielecka-Piontek

**Affiliations:** 1 Synteza sp. z o.o., św. Michała 67/71, 61-005 Poznań, Poland; magdalena.janczura@synteza.com.pl (M.J.); malgorzata.kobus-moryson@synteza.com.pl (M.K.-M.); 2 Department of Pharmacognosy, Faculty of Pharmacy, Poznań University of Medical Sciences, Święcickiego 4, 60-780 Poznań, Poland; szymonsip@ump.edu.pl; 3 Department of Developmental Neurology, Poznan University of Medical Sciences, Przybyszewski 49 Str., 60-355 Poznań, Poland; zarowski@ump.edu.pl; 4 Clinic for Rehabilitation, Poznan University of Medical Sciences, 28 Czerwca 1956r. nr 135/147 Street, 61-545 Poznań, Poland; agnieszka.warenczak@ump.edu.pl

**Keywords:** fixed-dose combination, polytherapy, pain pharmacotherapy, NSAIDs, spasmolytic drugs, painkillers, synergistic effect, abdominal pain, dysmenorrhea, innovations

## Abstract

This review presents the most common disease entities in which combinations of NSAIDs and spasmolytic drugs are used to reduce pain. The benefits of fixed-dose combination products (FDCs) are that they improve the response in people with insufficient monotherapy. Using the synergy or additive effect of drugs, it is possible to obtain a significant therapeutic effect and faster action with the use of smaller doses of individual drugs. In addition, one active ingredient may counteract adverse reactions from the other. Another essential aspect of the use of FDCs is the improvement of medical adherence due to the reduction in the pill burden on patients. It is also possible to develop a fixed-dosed combination product de novo to address a new therapeutic claim and be protected by patents so that the manufacturer can obtain exclusive rights to sell a particular FDC or a formulation thereof. The proposed fixed-dose combinations should always be based on valid therapeutic principles and consider the combined safety profile of all active substances included in the medicinal product. This review aims to identify which combinations of NSAIDs and spasmolytics have been developed and tested and which combinations are still under development.

## 1. Introduction

The pharmacotherapy of pain is often based on the need for polytherapy. There are many advantages of combined pain pharmacotherapy, including, above all, the possibility of obtaining an additive or synergistic effect. On the other hand, combinations allow one to increase the therapeutic effect while reducing the risk of drug-related side effects and using lower doses of individual drugs [1,2,3,4].

Despite the considerable progress of pharmacotherapy and the introduction of many new drugs, NSAIDs are still widely used and occupy an important place in the broadly understood pain management strategy [5,6]. NSAIDs are considered to be on the first rung of the analgesic ladder [7]. As painkillers, they are indicated, among others, in post-traumatic and muscular pains, pains after surgery or tooth extraction, neuralgia, root syndromes, discopathy, renal and hepatic colic, cancer pain, migraine, and menstrual pain [8,9,10,11,12]. When using NSAIDs to treat pain, it should be remembered that they exhibit a ceiling effect—producing no more substantial analgesic effect after exceeding a specific dose, usually the maximum dose recommended in the summary of product characteristics [13]. Therefore, a mistake is considered to be a situation in which the maximum amount of the drug is increased beyond the dose, instead of taking a drug with a more potent analgesic effect (e.g., weak opioid) or combining NSAIDs with a painkiller with a different gripping point (e.g., paracetamol) or with a spasmolytic agent [14,15,16,17].

However, special attention should also be paid to the limitations of the use of NSAIDs despite their frequent use. Particular restrictions on use are relevant for the elderly population, people taking anticoagulants, and people with a history of gastrointestinal bleeding and upper gastrointestinal ulcers, as NSAIDs are associated with frequent upper gastrointestinal bleeding [18,19,20]. In addition, NSAIDs should be used with caution in people with cardiovascular disease and have been found to tend to worsen congestive heart failure, increase blood pressure, and worsen adverse cardiovascular events, except acetylsalicylic acid, which is effective at low doses as a cardioprotectant [21,22]. Liver damage is a relatively rare case of side effects caused by NSAIDs; however, drugs from this group should not be used in the presence of cirrhosis of the liver due to the possibility of increased bleeding and the possibility of renal failure [23,24]. Most NSAIDs are considered safe in pregnancy, but their use is not recommended in the last 6–8 weeks; they may prolong pregnancy by inhibiting prostaglandin synthesis and problems that may arise in the fetus and mother due to reduce the production of platelets and disrupt clotting processes [25]. In addition, they should be used with caution in asthmatics; the possibility of causing or exacerbating the course of the disease has been demonstrated [26].

Spasmolytic drugs are characterized by side effects from the parasympathetic system [27,28]. The main side effects include nausea, dry mouth, itchy rashes, dizziness, breathing problems, headache, and increased pulse rate [29,30]. Hyoscine butylbromide should not be used in patients with myasthenia gravis [31]. Drotaverine hydrochloride should not be used in people with liver and kidney failure and in severe heart failure and in children under 12 years of age [32]. There have been no detailed clinical studies on the use of antispasmodics during pregnancy; due to the lack of clinical information, their use is not recommended, but they are not considered teratogenic. It is recommended not to use them in the first trimester and around delivery due to the possibility of accelerating labor [33].

The available scientific studies confirm the benefits of the combined administration of antispasmodic drugs and non-steroidal anti-inflammatory agents (NSAIDs) with analgesics and in the treatment of pain accompanying smooth muscle spasms of the urogenital system (renal colic, dysmenorrhea), gastrointestinal tract (intestinal colic, irritable bowel syndrome) and pathways cholecystitis (cholecystitis, cholangitis) [34,35,36,37].

## 2. Data Sources and Study Selection

The search for publications on combination drugs for pain therapy containing NSAIDs in combination with substances with spasmolytic activity was based on a selection of those scientific reports that included the assessment of their efficacy and safety.

Scientific literature and bibliographic databaseswere searched:

- The Medline;
- PubMed^®^;
- The Science Direct;
- The Cochrane Library (a database with systematic reviews).

Moreover, the following were checked:

- CENTRAL clinical trials registry;
- Drugs.com—an international drug information database;
- DrugBank—an international drug information database. The database is used by scientists and healthcare professionals around the world.

In the first stage, publications were searched automatically using keywords and related logical terms and then selected manually into the basic set.

The search keywords were: fixed-dose combination, polytherapy of pain, combined administration NSAIDs with spasmolytic agents; spasmolytic drugs; antispasmodic effects; combined pain pharmacotherapy; synergy or additive effects of drugs; combination drug with a different mechanism of action; ibuprofen plus drotaverine/alverine; metamizole and hyoscine combination; compare ibuprofen with drotaverine/alverine/hyoscine; ibuprofen plus drotaverine bilayer tablet; addition of spasmolytic agent; painkillers; pain relievers; two-component drugs; muscle relaxant and analgesic effect; multimodal analgesia; adjuvant; spastic pain during colic; abdominal pain; acute infectious gastroenteritis; menstrual pain; dysmenorrhea; alverine; drotaverine; hyoscine; pharmaceutical composition comprising two substances.

In addition, publications and patent applications containing commercial trade names of drugs were searched for: Buscopan compositum; Spasmofen; Petro tablets; Algopyrin complex; Quarelin; Drotin-M tablet; Algoflex-M, Vemonis Femi and their counterparts in other countries.

In stage 2, the following were excluded from the basic set:

- Publications not related to combinations of NSAIDs with other substances/combination drugs/the simultaneous administration of drugs with different mechanisms of action, pain polytherapy (publications on monotherapy were excluded);
- Publications on combinations other than combinations of NSAIDs with substances with a spasmolytic/analgesic effect;
- Publications that are too general and imprecise in describing conditions that are indications for using combinations of the listed substances;
- Publications published before 1980;
- Publications with the description of studies without randomization/placebo/blinding (not meeting the GCP requirements).

In the third stage, selected publications were qualified according to the selection criteria used. The publications included in the review were selected based on randomization, placebo, double-blind, or single-blind presence in the trial. They were carried out following the proper clinical trial (GCP) principles and developed based on the Helsinki Declaration and met medical, bioethical, and scientific research standards in research involving humans.

Most of the literature data used in this review relate to studies that meet the criteria and standards of clinical research following the GCP requirements. However, single studies were conducted before the introduction of the current GCP regulations.

The main aim of the review was to collect and include publications relating to clinical trials on the efficacy and tolerability of the concurrent use of NSAIDs and spasmolytic substances in the treatment of pain.

In stage 4, the qualified references were grouped into datasets, in which individual clinical data were assessed using weighting indicators related to selected selection criteria (Randomization (R), Blindness (Z), Clinical effect (E), Serious adverse reaction (C))

From 42 references, 21 clinical trials meeting this efficacy criterion were described. The report also presents 12 systematic reviews (meta-analyses) confirming the effectiveness of using NSAIDs in combination with other substances in various disease entities, including one review in the form of an expert opinion emphasizing that there are justified, both pharmacological and practical reasons for using combinations of drugs from the group of NSAIDs for pain management.

Fourteen compositions of registered, commercially available combination drugs containing a combination of NSAIDs and spasmolytic substances were identified and tabulated, along with commercial names and national availability, and innovative combinations under development/research.

## 3. Pharmacotherapy of Pain in Disease Entities

### 3.1. Acute Renal Colic Pain

The typical characteristic of renal colic is a sudden, unexpected pain radiating from the groin area, the immediate treatment of which in emergency departments is mainly based on rapid pain relief. Spasmofen contains 100 mg of ketoprofen and 10 mg of hyoscine butylbromide, and it is registered by Amriya Pharmaceutical Industries (Alexandria, Egypt) in the form of rectal suppositories. This combination is used to relieve severe colic pain quickly and effectively in the renal, hepatobiliary, or gastrointestinal tract. A study by Yakoto in patients who experienced acute renal colic compared using a single dose of Spasmofen rectal suppositories to the administration of a single intravenous (IV) dose of ketorolac tromethamine 30 mg/2 mL. The efficacy of therapy was noted after 60 min in 87.5% of patients treated with Spasmofen and 82.5% of patients treated with ketorolac. However, in the Fisher test, the difference was not statistically significant (*p* = 0.755). The mean percentage reduction in VPAS at 15 min post-dose was 61.82% in the Spasmofen group and 64.76% in the ketorolac group. In the conducted study, within one hour from administering the therapeutic dose, Spasmofen was related with a statistically significant greater reduction in VPAS (mean% reduction = 92.36%) than ketorolac (75.06%; *p* = 0.0466). Combining an antispasmodic agent with a potent analgesic in the form of a rectal suppository with a good bioavailability profile, although it reaches its maximum effect to some extent slower than when administered intravenously, can provide high effectiveness in reducing pain in renal colic by operating based on two separate mechanisms of action. Spaspofen as a fixed-dose drug, in the form of a rectal suppository, can be a safe, rapid, and effective method of treating acute renal colic in emergency departments [38].

Sarfraz et al. performed a study of the effectiveness of the combination of hyoscine butylbromide with diclofenac compared to the action of diclofenac alone; the obtained results showed significantly higher effectiveness of the use of the combination therapy compared to the use of diclofenac alone in the treatment of pain caused by renal colic. Patients on monotherapy received a single dose of 75 mg of diclofenac sodium as an intramuscular injection. The second study group treated with the combination therapy in addition to receiving an intramuscular injection of 75 mg of diclofenac sodium received a single dose of 20 mg of hyoscine-N-butyl bromide as an intravenous injection. A significantly greater number of patients were observed in the conducted study whose pain caused by renal colic was relieved after 30 min after administering diclofenac and hyoscine combined with those treated only with diclofenac. There was no significant difference between the two treatment groups at 60 min after drug treatment. The obtained results indicate the advantage of using the combined therapy of diclofenac with hyoscine over diclofenac alone in pain control in patients with renal colic due to the need to obtain an immediate and prolonged analgesic effect [39].

Ali’s study compared the effects of tenoxicam (NSAID) with buscopan. The results obtained showed that 80% of patients treated with tenoxicam achieved pain relief after one hour compared to 86% of patients in this study [40].

A study by Kumar et al. aimed to compare the therapeutic effect of using diclofenac and hyoscine in the symptomatic treatment of acute colic. The study showed that 91.7% of patients treated with diclofenac achieved complete pain relief one hour after dosing. However, the studies mentioned above were designed to compare the effect of NSAIDs and hyoscine alone, not as a combination; the results obtained by Ali and Kumar et al. indicate pain relief one hour after treatment in 72.7% and 69.4% of patients treated with hyoscine alone [40,41].

Combining hyoscine butylbromide with analgesics such as ibuprofen, acetaminophen, and metamizole sodium (Buscopan Compositum, Boehringer Ingelheim) has already been marketed in some countries for a decade.

It is common in clinical practice to use a combination of NSAID drugs with antispasmodics. Application in the form of injections of NSAIDs and antispasmodics in acute renal colic helps relieve pain while reducing inflammation and swelling at the site of ureteral obstruction. The addition of antispasmodics has been shown to increase the effectiveness of NSAID treatment in acute spasmodic pain. However, the literature on the subject shows that few published studies deal with the safety and effectiveness of such a combination and the advantage of using one of them. Porwal et al. compared dicyclomine combinations with other NSAIDs and showed different efficacy in the acute pain model. The study compared diclofenac and dicyclomine administered intravenously with the combination of dexketoprofen and dicyclomine to treat acute renal colic. The researchers found that the second combination was much more effective and better tolerated than the first drug combination.

In this study, a total of 270 patients were randomized to either use a combination of dexketoprofen and dicyclomine intramuscularly (*n* = 109) or a fixed-dose combination of diclofenac and dicyclomine (*n* = 108). The pain intensity was assessed using a visual analog scale (VAS) for eight hours at two-hour intervals. The conducted study showed that the product containing dexketoprofen and dicyclomine showed better efficacy and better tolerance than the fixed-dose combination of diclofenac and dicyclomine in patients diagnosed with acute renal colic when administered intravenously [42].

### 3.2. Recurrent Crampy Abdominal Pain

It is estimated that cramping abdominal pain affects up to 30% of the adult population in Western countries. It is considered a functional disorder of the digestive tract after excluding an organic, parasitic, or infectious disease. Therefore, the primary symptomatic treatment is based on the administration of antispasmodics to patients with recurrent crampy abdominal pain.

A study by Mueller-Lissner et al. compared the efficacy and tolerability usage of 10 mg of hyoscine butylbromide and 500 mg of paracetamol and their fixed-dose combination compared to a placebo in recurrent cramping abdominal pain. A total of 1637 patients were randomized to one of the four treatment groups after a one-week placebo run-in period. The treatment lasted for three weeks, with a rating in the first, second, and third week. The study included a daily determination of the pain intensity (visual analog scale) and frequency (verbal rating scale) of its occurrence. The study showed that hyoscine, paracetamol, and their fixed combination effectively treat recurrent cramping abdominal pain and are well tolerated when administered three times a day continuously for three weeks [43].

### 3.3. Abdominal Pain in Acute Infectious Gastroenteritis

A randomized controlled study by Narang was designed to evaluate the efficacy and safety of a combination of 500 mg of paracetamol with 80 mg of drotaverine hydrochloride in the treatment of abdominal pain caused by acute infectious gastroenteritis. The study included a total of 252 adults of either gender with acute infectious diarrhea and moderate-to-severe abdominal pain. Patients were treated with a fixed-dose combination of 500 mg of paracetamol combined with 80 mg of drotaverine hydrochloride or with paracetamol alone in the same dose; drugs were administered three times daily for three days. The mean intensity of the pain difference was observed at 60 min after applying the therapeutic dose and complete pain relief after two hours using the VAS and Verbal Rating Scale.

The conducted study showed a statistically significant improvement in using the combination of drotaverine hydrochloride with paracetamol compared to paracetamol alone. Moreover, much faster pain relief was observed in the group treated with the fixed-dose combination [44].

### 3.4. Menstrual Pain

Menstrual pain is a visceral, or diffuse, poorly localized pain with a component of inflammatory pain. The most likely cause of menstrual pain is currently considered to be excessive uterine contractile function due to increased levels of prostaglandins, increased sympathetic tone, and reduced uterine blood flow. The painkillers of choice for women with primary dysmenorrhea are non-steroidal anti-inflammatory drugs (NSAIDs) that reduce prostaglandin synthesis. In patients treated with NSAIDs, including ibuprofen, the levels of prostaglandins in menstrual secretions are reduced [45]. The non-steroidal prostaglandin synthetase inhibitors are administered from the beginning of menstruation for several days. Nimesulide (2 × 100 mg), ketoprofen (2 × 100 mg), mefenamic acid (3 × 500 mg), naproxen (3 × 250–500 mg) or ibuprofen (3 × 200–400 mg) are particularly recommended. These drugs are best taken with meals. It should be remembered that it is inadvisable to administer two different NSAIDs at the same time. For women with premenstrual syndrome (PMS), medication can be given just before the onset of menstruation (pain relief) [46].

This type of therapy is effective for approximately 80% of patients. Non-steroidal anti-inflammatory drugs (NSAIDs), which include ibuprofen, act causally in primary therapy dysmenorrhea. Women with primary dysmenorrhea often use NSAIDs in very high doses, exposing themselves to numerous side effects, i.e., irritation of the gastric mucosa, leading to stomach ulcers, nausea, vomiting, and nervous system disorders [45].

In a randomized trial, Dawood demonstrated the greater efficacy of ketoprofen at 12.5 and 25 mg and ibuprofen at 200 mg than a placebo, with a complete recovery of pain at the fourth hour after administering both test drugs [46]. In addition, Rofecoxib, a selective COX-2 inhibitor, has proved to be a very effective drug when treating primary dysmenorrhea [47]. However, due to the increased risk of causing complications within the system, cardiovascular disease (heart attack, stroke) has been withdrawn from the market, similar to other drugs from this group (e.g., valdecoxib) [48].

Given the mechanism of prostaglandin-dependent pain, it can be argued that non-steroidal anti-inflammatory drugs (NSAIDs), that act by inhibiting COX-1 and COX-2, create the ideal therapeutic path. However, the pathomechanism of menstrual pain is more complex and is used in many inhibitions of PG production, which does not cause pain relief. The use of additional cyclooxygenase two inhibitors (Coxibs), despite initial hopes, did not bring the expected results. Two independent studies confirmed the lack of superiority of selective COX-2 inhibitors over non-selective NSAIDs in treating the effects of menstrual pain [47,48]. The treatment of visceral pain, with a component of inflammatory pain, used not only non-steroidal anti-inflammatory drugs, but also spasmolytics, acting on the uterine muscle contraction. If monotherapy fails, it is recommended to use a combination of two drugs: NSAID and metamizole, which has a powerful spasmolytic effect (metamizole dose up to 5 × 1 g), or NSAID and drotaverine (up to 3 × 80 mg), or NSAID and alverine (in a dose of up to 3 × 120 mg). 

Özbay assessed the efficacy of lornoxicam, alverine citrate + simethicone versus a placebo in treating dysmenorrhea. The study included 33 patients with primary dysmenorrhoea who took lornoxicam alverine citrate with simethicone and a placebo for two cycles. The pain reduction efficacy was assessed using the visual analog scale (VAS). In the results, the placebo was found ineffective (*p* = 0.989), lornoxicam and alverine citrate were determined to effectively treat primary dysmenorrhea (*p* values, respectively: *p* = 0.000, *p* = 0.001). Thus, the presented results are consistent with the literature on the subject, supporting the use of NSAIDs in the treatment of primary dysmenorrhea. In addition, alverine citrate, which has a smooth muscle relaxant and analgesic effect, appears to be helpful as an adjunct to the treatment of primary dysmenorrhea. However, more advanced and extensive research is required to accurately determine the effect of alverine citrate on primary dysmenorrhea [49].

Drotaverine, like alverine, is a derivative of papaverine. Drotaverine, marketed under the No-spa^®^ trade name, is a popular antispasmodic drug in some countries in eastern and Central Europe and sub-Saharan Africa. The unique combination of ibuprofen and drotaverine drug was developed and patented by Sanofi-Aventis to treat menstrual pain and abdominal cramps. The two-component drug contains 400 mg of ibuprofen and 80 mg of drotaverine hydrochloride, as a combination pack, comprising six tablets of each. This product is sold under the Algoflex-M^®^ trademark in some European countries such as Hungary [34]. Dębski et al. conducted an assessment of the effectiveness and tolerability of 80 mg of drotaverine and 400 mg of ibuprofen in patients with primary dysmenorrhea—the DOROTA study. The weighted sum of pain intensity differences was calculated based on the six-hour observation time (SPID-6). Before the test, the intensity of pain in both groups was assessed as comparable. However, at subsequent specified time points, the reduction in pain was more significant in the study group treated with 400 mg of ibuprofen than in the group treated with 80 mg of drotaverine, and was complete at 4 h in the drotaverine and 3 h in the ibuprofen group. Thus, 41.8% of patients treated with drotaverine and 68.6% treated with ibuprofen evaluated drug efficacy as excellent or good. However, the patient’s global assessment of tolerability was significantly better (*p* = 0.02) with ibuprofen (400 mg) (86.8%) than with drotaverine (80 mg) (78.4%) [50].

The ternary drug, containing metamizole, drotaverine, and caffeine, is an example of a well-established FDC with potent analgesic and antipyretic properties. The combination of analgesic (metamizole) with antispasmodic (drotaverine) effectively relieves persistent spastic pain during colic, abdominal pain, menstruation, migraine, etc. Caffeine is an adjuvant, commonly used as an adjunct to over-the-counter pain relievers. An adjuvant is a compound that is added to a drug to increase its effectiveness. A study compared the effects of ibuprofen at 400 mg and ibuprofen at the same dose combined with 100 mg of caffeine in patients who had their third molar tooth extracted. The superiority of the two-component preparation over ibuprofen alone was observed in the drug efficacy and speed of action. A review of four studies suggests that a lower dose of ibuprofen (200 mg) combined with 100 mg of caffeine is more effective in treating post-operative pain [51,52]. The commercially available drugs containing metamizole, drotaverine, and caffeine are Petro tablets from Alpha Chem (Cairo, Egypt) and Vemonis Femi^®^ from Adamed Pharma (Pieńków, Poland). Unfortunately, a combination of the above-mentioned medicinal substances, which is the only one available on the European market, fails to meet the chemical purity requirements. The chemical quality deteriorates even further over time, during the medicine storage, as the impurities increase due to the critical active components. Attention should be paid in particular to the high level of impurities of drotaverine derivatives, the limit of which, for a single unknown impurity, is 0.2%. It is, therefore, desirable to provide a pharmaceutical composition comprising these three active substances with good stability or use another combination with a similar spectrum of action [53].

### 3.5. Labor

In the last decade, we have observed a high frequency of using spasmolytics and combinations of spasm-analgesics to facilitate cervical dilatation during labor and shorten the first phase and the entire duration of labor. However, anticholinergic spasmolytic drugs have numerous undesirable side effects that can lead to dangerous situations for both the mother and the fetus. Hence, non-anticholinergic spasmolytics are nowadays the preferable choice during pregnancy. Atropine, when given by injection to a woman during pregnancy, increases the fetal heart rate. Dicyclomine has been linked with a few cases of human congenital disabilities. When injected into humans during pregnancy, hyoscyamine increases the fetal heartbeat. The desired antispasmodics are rapid action and long-lasting, do not adversely affect the uterine contractions, and do not risk uterine inertia. They also have minimal side effects on the mother and fetus. More than a few studies focused on various antispasmodic agents to shorten the phase of labor, among which are hyoscine-N-butyl bromide, drotaverine hydrochloride, phloroglucinol, and valethamate bromide.

A prospective randomized trial of 146 women in active, spontaneous labor was conducted by Madhu et al. The patients were assigned to three equal groups, in which women were given drotaverine or valethamate, and the control group was given a placebo. According to the protocol, the amniotomy was performed with a 4 cm dilation of the cervix. There was a statistically significant difference in the meantime from intravenous administration to delivery: 183.2 min in the drotaverine group, compared to 206.5 min in the valethamate group and 245 min in the control group. The mean cervical dilatation factor (cm/h) was statistically significant and amounted to 3, 2.4, and 1.9 (in the groups: 1, 2, 3). There were no statistically significant differences in the duration of the second and third stages of labor. Short-lived side effects, such as fetal–maternal tachycardia, dryness of mouth, and flushing of the face, were noted for valethamate. Both drotaverine and valethamate appear to help cervical dilatation and augment the first stage of labor significantly. However, drotaverine accelerates labor more rapidly and is associated with fewer side effects than valethamate [54].

In the conducted study by Ibrahim et al., the role of antispasmodic drug drotaverine in shortening the length of the active first stage of labor among nulliparous women was assessed. At the start of the active phase of labor, drotaverine hydrochloride (40 mg) was administered to one group and placebo to the other group. Patients received a maximum of three doses of the drug, separated by 2 h. There was a significant difference in the assessment of labor pain after the administration of drotaverine, the active phase of labor duration, and the cervical dilatation index between the two groups (*p* < 0.001 for all). In this study, no maternal differences in adverse effects were observed. Kaplan–Meier survival analysis showed a higher probability of swifter delivery among patients treated by drotaverine hydrochloride (log-rank test; *p* < 0.001). Drotaverine hydrochloride effectively and safely shortened the first stage of labor among nulliparous women with active, spontaneous labor [55].

The mechanism of NSAIDs in labor pain reduction is carried out by suppressing the production of prostaglandins that cause inflammation and pain. In the periphery, NSAIDs work by reducing the sensitivity of the nociceptor (sensory neurons responsible for feeling pain externally or internally) to painful stimuli induced by heat, trauma, or inflammation.

The use of diclofenac for pain relief in the first stage of labor was associated with a statistically significant reduction in pain scores in both primiparous and multiparous women. However, there was no statistically significant effect of diclofenac administration on the duration of the first and second stages of labor in primiparous and multiparous women, nor were the neonatal or maternal side effects detected [56].

The analgesic efficacy of aceclofenac was evaluated compared to paracetamol in women undergoing episiotomy during labor. A dose of 100 mg of aceclofenac has been shown to be more potent than 650 mg of paracetamol in providing relief from post episiotomy pain, particularly 3 to 5 h after administration. The patients’ severity was assessed using an analog visual test (Huskisson’s test). The antalgic effect of paracetamol showed a similar evolution over time to aceclofenac, but the analgesic efficacy of paracetamol seemed to be much less [57].

The current review presented by Tiwari gives the foundation for selecting an effective combination of aceclofenac (a COX-2 inhibitor) and drotaverine hydrochloride (antispasmodic agent with non-anticholinergic action) as an appropriate choice to amplify the labor over the other proposed combinations [48]. However, large, strict, randomized controlled trials are needed to estimate the effect of antispasmodics on prolonged labor and evaluate their effect on labor in the expectant management of labor.

## 4. Developed Combinations of NSAID and Antispasmodic Agents

There are several combinations of NSAID and antispasmodic agents available in the market or under research. The characteristics of given fixed-dose combinations are presented in Table 1.

## 5. Conclusions

A patient’s response to therapy cannot be fully predicted from the drug’s mechanism of action alone. No therapy is optimal for all patients. Therefore, the assessment of effectiveness is only possible based on the observation of individual patients. The combination of various painkillers and adjuvants with different action mechanisms can help optimize the analgesic effect. The implementation of standard pain assessment, the routine use of multimodal analgesia techniques, and the development of combination drugs for pain management are signs promising the continuation of the improvement of pain management in the coming years. Despite the research potential, surprisingly few controlled studies have been conducted using spasmolytic drugs combined with analgesics to treat cramp pain. A small number of drugs are available on the Polish market containing a combination of NSAIDs and spasmolytic substances. Very few combinations of analgesics and antispasmodic agents are available to treat menstrual pains and cramps in Europe. When developing new formulations, particular attention should be paid to the possibility of using the given active compounds in as many countries as possible; for example, drotaverine is not approved for use by the FDA, which significantly reduces the potential market.

Moreover, these are available as two different tablets in a single pack. Hence, to provide a compact formulation for patient compliance, there is still a need to prepare a fixed-dose combination of NSAIDs and spasmolytic agents available in an easy-to-administer single tablet dosage form. It is, therefore, desirable to provide a pharmaceutical composition comprising this combination of active substances with good stability. Particular attention should also be paid to the need to perform additional detailed clinical trials aimed at determining the effectiveness of therapy using fixed-dose combinations and determining their safe profile of action. The risk of adverse effects always increases with the number of drugs used; therefore, it seems necessary to perform additional clinical trials before admitting new formulations to the market to verify the safety of use.

This review aims to indicate which combinations of NSAIDs and spasmolytics have been developed and tested and which combinations are still under development. It shows a broad potential for future research to develop modern, effective combinations and search for new indications for the existing fixed-dose combination drugs.

## Figures and Tables

**Table 1:** Developed combinations of NSAID and antispasmodic agents.

	Marketed Products	Combination of Drugs	Manufacturer	Effect of Drug
1	Drotin Plus Tablet	Drotaverine 80 mg + Paracetamol 500 mg	Walter Bushnell, India	Fixed-dose combination used in the treatment of abdominal pain. It effectively reduces abdominal pain, bloating, discomfort, and cramps by relaxing the muscles of the stomach and gut. It also alters chemical signaling that causes pain and fever.
	Verin P		Corona Remedies Pvt Ltd., India	
	Esnil P		Dewcare Concept Pvt. Ltd.	
	Travin P		India Biometrix Pharma, India	
2	Drotin-M Tablet	Drotaverine 80 mg + Mefenamic Acid 250 mg	Walter Bushnell, India	Drotaverine HCl antispasmodic agent with non-anticholinergic action. The Tuela Tablet is used for functional bowel disorders, pain in renal colic, analgesic, and other conditions.
	Tuela Tablet		Cipla Ltd., India	
3	Almefkem Spas	Dicycloverine 10 mg + Mefenamic Acid 250 mg	Alkem, India	Medicine is used to provide symptomatic relief from menstrual (period-related) pain and cramps. It is also used to treat abdominal pain by relieving spasms of the muscles in the stomach and intestines.
	Mef-Proxyvon		Wockhardt, India	
	Antspas		Absolute, India	
	Paraspas		Cadila Pharmaceuticals Ltd., India	
4	Trigan MF	Dicycloverine 20 mg + Paracetamol 500 mg + Mefenamic Acid 250 mg	Cadila Pharmaceuticals Ltd., India	The Trigan MF Tablet is a fixed-dose medicine used in the treatment of abdominal cramps. It effectively reduces abdominal pain and cramps by relaxing the muscles of the stomach and gut. It also alters chemical signaling that causes pain and fever.
5	Spasmofen ampoules	Hyoscine butylbromide 20 mg + Ketoprofen 100 mg	Amriya Pharmaceuticals Co., Egypt.	Spasmofen is indicated in acute spasm, as in renal or biliary colic, in radiology for the differential diagnosis of obstruction, reduced spasm and pain in pyelography, and other diagnostic procedures where spasms may be a problem c gastro-duodenal endoscopy.
6	Orcigesic tablet	Orphenadrine citrate 35 mg + Mefenamic acid 250 mg	Sunward Pharmaceutical, Singapore	The Orcigesic Tablet is used for acute painful musculoskeletal conditions, febrility, muscle pain, analgesic, pain during periods, heavy bleeding during periods, fever, inflammation, migraine headache, tooth pain, and other conditions. Orphenadrine is a muscarinic antagonist with muscle relaxant activity. Mefenamic acid is a non-steroidal anti-inflammatory agent with analgesic, anti-inflammatory and antipyretic properties. It is an inhibitor of cyclooxygenase.
7	Norgesic/Norgesic forte	Orphenadrine citrate 50 mg + Aspirin 770 mg + Caffeine 60 mg.	Riker Laboratories, United States	Norgesic has a combination of analgesic, muscle relaxant, anti-inflammatory, and antipyretic properties. Orphenadrine citrate is a centrally acting brain stem compound with analgesic and some anticholinergic properties. It selectively blocks facilitatory functions of the reticular formation. Norgesic is indicated for the symptomatic relief of the mild to moderate pain of acute musculoskeletal disorders. discontinued by the FDA in the USA in October 2015, still available in Scandinavia, Austria, Greece, Thailand, Australia and Hong Kong.
8	Fast Free	Hyoscine butylbromide 10 mg + Ketoprofen 100 mg	Delta Pharma, Egypt	This combination is intended for the swift relief of severe colicky pain in the renal system, hepatobiliary system, or gastrointestinal tract.
	Spasmofen (Rectal Suppositories)		Amriya, Egypt	
9	Dolorsin Complex	Hyoscine butylbromide 20 mg + Ibuprofen, 400 mg + Caffeine 50 mg	Novamed, Colombia	Analgesic and antispasmodic useful in the treatment of strong and painful uterine spasms associated with the dysmenorrhea.
10	Ibualpin Ella	Hyoscine butylbromide 20 mg + Ibuprofen 400 mg	Alpifar, Paraguay	Hyoscine with ibuprofen is indicated for acute spasms of the gastrointestinal tract. Spasm and biliary dyskinesia. Spasm of the genitourinary tract.
	Ibuflam Fem		Incopharma, Paraguay	
	Compofen		Boehringer Ingelheim, Colombia	
11	Scopolan Compositum	Hyoscine butylbromide 10 mg + Metamizole 250 mg.	Herbapol Wrocław, Poland	Indicated for the short-term treatment of severe pain in spasms: - gastrointestinal tract (e.g., stomach cramps, intestinal colic, irritable bowel syndrome), - biliary tract (e.g., biliary colic), - the genitourinary system (e.g., renal colic, urolithiasis associated with ureterolithiasis, dysmenorrhoea).
	Buscopan Compositum		Boehringer Ingelheim	
12	Panadol Femina	Hyoscine butylbromide 10 mg + Paracetamol 500 mg	Glaxo Wellcome Poznan, Poland	Acetaminophen is used to relieve pain and decrease fever. Hyoscine butylbromide tolerates gastrointestinal and urogenital spasms. The preparation is used to prevent and treat ailments related to dysmenorrhea, kidney or liver colic, irritable bowel syndrome.
	Buscopan Compositum		Boehringer Ingelheim	
13	Algopyrin Complex,	Metamizole 400 mg + Drotaverine hydrochloride 40 mg + Caffeine 60 mg	Sanofi-Aventis Hungary	Intended for use in adults for the symptomatic treatment of: - severe pain of various origins - pain associated with smooth muscle spasms: - the genitourinary system (renal colic, dysmenorrhea), - gastrointestinal tract (intestinal colic, irritable bowel syndrome), - biliary tract (cholecystitis, cholangitis), when the use of other drugs is contraindicated or ineffective.
	Quarelin		Sanofi-Aventis Hungary	
	Petro Tablets		Alpha Chem Advanced Pharmaceutical CO. (ACAPI)	
	Vemonis Femi		Adamed Pharma S.A, Poland	
14	Algoflex-M	Ibuprofen 400 mg + Drotaverine hydrochloride 80 mg (as a combination pack comprising six tablets each; single tablet dosage form not available)	Sanofi-Aventis, Hungary	Algoflex-M tablets—painkillers, anti-inflammatory, and antispasmodic medicines are used to treat primary (not explained by other diseases) menstrual pain and abdominal cramps and adjuncts the treatment of secondary menstrual cramps and abdominal cramps.
15	Not Available	Piroxicam/Meloxicam + Phloroglucinol/Trimetylphloroglucinol	Under research [58]	The composition, which is used to control and treat renal colic and inflammation caused by lithiasis, provides effective pain control and ensures that renal function remains healthy and suppresses and alleviates the effects of urethral obstruction.
16	Not Available	Alverine citrate + Ibuprofen/Lornoxicam	Under research	This combination is intended for the rapid relief of severe colicky pain in the renal system, hepatobiliary system, gastrointestinal tract or dysmenorrhea.

## References

[1] Stasiłowicz A., Tykarska E., Rosiak N., Sałat K., Furgała-Wojas A., Plech T., Lewandowska K., Pikosz K., Pawłowicz K., Cielecka-Piontek J. (2021). The Inclusion of Tolfenamic Acid into Cyclodextrins Stimulated by Microenvironmental PH Modification as a Way to Increase the Anti-Migraine Effect. JPR.

[2] Shah D.D., Sorathia Z.H. (2020). Tramadol/Diclofenac Fixed-Dose Combination: A Review of Its Use in Severe Acute Pain. Pain Ther..

[3] Schulte H., Sollevi A., Segerdahl M. (2004). The Synergistic Effect of Combined Treatment with Systemic Ketamine and Morphine on Experimentally Induced Windup-like Pain in Humans. Anesth. Analg..

[4] Raffa R.B., Tallarida R.J., Taylor R., Pergolizzi J.V. (2012). Fixed-Dose Combinations for Emerging Treatment of Pain. Expert Opin. Pharmacother..

[5] Wongrakpanich S., Wongrakpanich A., Melhado K., Rangaswami J. (2018). A Comprehensive Review of Non-Steroidal Anti-Inflammatory Drug Use in The Elderly. Aging Dis..

[6] Peterson K., McDonagh M., Thakurta S., Dana T., Roberts C., Chou R., Helfand M. (2010). Drug Class Review: Non-Steroidal Anti-inflammatory Drugs (NSAIDs): Final Update 4 Report.

[7] Anekar A.A., Cascella M. (2021). WHO Analgesic Ladder. StatPearls.

[8] Rosenbloom D., Craven M.A. (1983). A Review of Non-Steroidal Anti-Inflammatory Drugs. Can. Fam. Physician.

[9] Abdu N., Mosazghi A., Teweldemedhin S., Asfaha L., Teshale M., Kibreab M., Anand I.S., Tesfamariam E.H., Russom M. (2020). Non-Steroidal Anti-Inflammatory Drugs (NSAIDs): Usage and Co-Prescription with Other Potentially Interacting Drugs in Elderly: A Cross-Sectional Study. PLoS ONE.

[10] Pardutz A., Schoenen J. (2010). NSAIDs in the Acute Treatment of Migraine: A Review of Clinical and Experimental Data. Pharmaceuticals.

[11] Hersh E.V., Moore P.A., Grosser T., Polomano R.C., Farrar J.T., Saraghi M., Juska S.A., Mitchell C.H., Theken K.N. (2020). Nonsteroidal Anti-Inflammatory Drugs and Opioids in Postsurgical Dental Pain. J. Dent. Res..

[12] Morelli K.M., Brown L.B., Warren G.L. (2018). Effect of NSAIDs on Recovery From Acute Skeletal Muscle Injury: A Systematic Review and Meta-Analysis. Am. J. Sports Med..

[13] Marjoribanks J., Ayeleke R.O., Farquhar C., Proctor M. (2015). Nonsteroidal Anti-Inflammatory Drugs for Dysmenorrhoea. Cochrane Database Syst. Rev..

[14] Hyllested M., Jones S., Pedersen J.L., Kehlet H. (2002). Comparative Effect of Paracetamol, NSAIDs or Their Combination in Post-operative Pain Management: A Qualitative Review. Br. J. Anaesth..

[15] Afshar K., Jafari S., Marks A.J., Eftekhari A., MacNeily A.E. (2015). Nonsteroidal Anti-Inflammatory Drugs (NSAIDs) and Non-Opioids for Acute Renal Colic. Cochrane Database Syst. Rev..

[16] Ong C.K.S., Seymour R.A., Lirk P., Merry A.F. (2010). Combining Paracetamol (Acetaminophen) with Non-steroidal Anti-inflammatory Drugs: A Qualitative Systematic Review of Analgesic Efficacy for Acute Postoperative Pain. Anesth. Analg..

[17] Thybo K.H., Hägi-Pedersen D., Wetterslev J., Dahl J.B., Schrøder H.M., Bülow H.H., Bjørck J.G., Mathiesen O. (2017). PANSAID–PAracetamol and NSAID in Combination: Study Protocol for a Randomised Trial. Trials.

[18] Risser A., Donovan D., Heintzman J., Page T. (2009). NSAID Prescribing Precautions. AFP.

[19] Tai F.W.D., McAlindon M.E. (2021). Non-Steroidal Anti-Inflammatory Drugs and the Gastrointestinal Tract. Clin. Med..

[20] Wan Ghazali W.S., Wan Zainudin W.M.K.B., Yahya N.K., Mohamed Ismail A., Wong K.K. (2021). Older Age and Diclofenac Are Associated with Increased Risk of Upper Gastrointestinal Bleeding in Gout Patients. PeerJ.

[21] Christiansen M., Grove E.L., Hvas A.-M. (2021). Contemporary Clinical Use of Aspirin: Mechanisms of Action, Current Concepts, Unresolved Questions, and Future Perspectives. Semin. Thromb. Hemost..

[22] Laoruengthana A., Chaibhuddanugul N., Rattanaprichavej P., Malisorn S., Tangsripong P., Pongpirul K. (2021). Perioperative Outcomes of Patients Who Were Not Candidates for Additional Non-steroidal Anti-Inflammatory Drugs in a Multimodal Pain Control Regimen for Total Knee Arthroplasty. Clin. Orthop. Surg..

[23] Ershad M., Ameer M.A., Vearrier D. (2021). Ibuprofen Toxicity. StatPearls.

[24] Lieber S.R., Jiang Y., Moon A., Barritt A.S. (2021). Antiplatelet Medications Are Associated With Bleeding and Decompensation Events Among Patients With Cirrhosis. J. Clin. Gastroenterol..

[25] Antonucci R., Zaffanello M., Puxeddu E., Porcella A., Cuzzolin L., Pilloni M.D., Fanos V. (2012). Use of Non-Steroidal Anti-Inflammatory Drugs in Pregnancy: Impact on the Fetus and Newborn. Curr. Drug. Metab..

[26] Lo P.-C., Tsai Y.-T., Lin S.-K., Lai J.-N. (2016). Risk of Asthma Exacerbation Associated with Non-steroidal Anti-Inflammatory Drugs in Childhood Asthma. Medicine.

[27] Lee H., Son H.J., Rhee P.-L., Kim J.J., Rhee J.C. (2007). Unexpected Anterograde Amnesia Associated with Buscopan Used as a Predmedication for Endocscopy. World J. Gastroenterol..

[28] Romics I., Molnár D.L., Timberg G., Mrklic B., Jelakovic B., Köszegi G., Blaskó G. (2003). The Effect of Drotaverine Hydrochloride in Acute Colicky Pain Caused by Renal and Ureteric Stones. BJU Internatl..

[29] Xue X., Qi X.-X., Wan X.-Y. (2017). Randomized Controlled Study of Efficacy and Safety of Drotaverine Hydrochloride in Patients with Irritable Bowel Syndrome. Medicine.

[30] Chen G.-L., Hsu W.-H. (2013). Hyoscine-N-Butyl-Bromide-Induced Hypotension and Myocardial Ischemia. Case Rep. Crit. Care.

[31] Dyde R., Chapman A.H., Gale R., Mackintosh A., Tolan D.J.M. (2008). Precautions to Be Taken by Radiologists and Radiographers When Prescribing Hyoscine-N-Butylbromide. Clin. Radiol..

[32] Woroń J., Filipczak-Bryniarska I., Wordliczek J. (2011). Leki złożone w farmakoterapii bólu. Paracetamol i tramadol. 4. Geriatria.

[33] Meyer A., Fermaut M., Drouin J., Carbonnel F., Weill A. (2021). Drug Use for Gastrointestinal Symptoms during Pregnancy: A French Nationwide Study 2010–2018. PLoS ONE.

[34] Colli A., Conte D., Valle S.D., Sciola V., Fraquelli M. (2012). Meta-Analysis: Non-steroidal Anti-Inflammatory Drugs in Biliary Colic. Aliment. Pharmacol. Ther..

[35] Inage K., Orita S., Yamauchi K., Suzuki T., Suzuki M., Sakuma Y., Kubota G., Oikawa Y., Sainoh T., Sato J. (2016). Low-Dose Tramadol and Non-Steroidal Anti-Inflammatory Drug Combination Therapy Prevents the Transition to Chronic Low Back Pain. Asian Spine J..

[36] Fendrick A.M., Greenberg B.P. (2009). A Review of the Benefits and Risks of Non-steroidal Anti-Inflammatory Drugs in the Management of Mild-to-Moderate Osteoarthritis. Osteopath Med. Prim. Care.

[37] Magee D.J., Jhanji S., Poulogiannis G., Farquhar-Smith P., Brown M.R.D. (2019). Non-steroidal Anti-Inflammatory Drugs and Pain in Cancer Patients: A Systematic Review and Reappraisal of the Evidence. Br. J. Anaesth..

[38] Yakoot M., Salem A., Yousef S., Helmy S. (2014). Clinical Efficacy of Spasmofen^®^ Suppository in the Emergency Treatment of Renal Colic: A Randomized, Double-Blind, Double-Dummy Comparative Trial. Drug Des. Devel. Ther..

[39] Sarfraz K., Shehzad K., Sheikh I.A. (2008). Comparison between Pain Relief Achieved by Diclofenac Alone and the Combination of Diclofenac and Hyoscine in the Short-Term Treatment of Ureteric Colic. PAFMJ.

[40] al-Waili N.S., Saloom K.Y. (1998). Intravenous Tenoxicam to Treat Acute Renal Colic: Comparison with Buscopan Compositum. J. Pak. Med. Assoc..

[41] Kumar A., Deed J.S., Bhasin B., Kumar A., Thomas S. (2004). Comparison of the Effect of Diclofenac with Hyoscine-N-Butylbromide in the Symptomatic Treatment of Acute Biliary Colic. ANZ J. Surg..

[42] Porwal A., Mahajan A.D., Oswal D.S., Erram S.S., Sheth D.N., Balamurugan S., Kamat V., Enadle R.P., Badadare A., Bhatnagar S.K. (2012). Efficacy and Tolerability of Fixed-Dose Combination of Dexketoprofen and Dicyclomine Injection in Acute Renal Colic. Pain Res. Treat..

[43] Mueller-Lissner S., Tytgat G.N., Paulo L.G., Quigley E.M.M., Bubeck J., Peil H., Schaefer E. (2006). Placebo—And Paracetamol-Controlled Study on the Efficacy and Tolerability of Hyoscine Butylbromide in the Treatment of Patients with Recurrent Crampy Abdominal Pain. Aliment Pharmacol. Ther..

[44] Narang S., Koli J. (2018). Efficacy and Safety of Fixed-Dose Combination of Drotaverine Hydrochloride (80 Mg) and Paracetamol (500 Mg) in Amelioration of Abdominal Pain in Acute Infectious Gastroenteritis: A Randomized Controlled Trial. J. Gastroenterol. Hepatol..

[45] Chan W.Y., Dawood M.Y., Fuchs F. (1979). Relief of Dysmenorrhea with the Prostaglandin Synthetase Inhibitor Ibuprofen: Effect on Prostaglandin Levels in Menstrual Fluid. Am. J. Obstet. Gynecol..

[46] Dawood M.Y. (1994). Multi-Center, Randomized Double-Blind, Crossover Study Comparing Ketoprofen 12.5 Mg and 25 Mg, Ibuprofen 200 Mg and Placebo in the Treatment of Primary Dysmenorrhea.

[47] Morrison B.W., Daniels S.E., Kotey P., Cantu N., Seidenberg B. (1999). Rofecoxib, a Specific Cyclooxygenase-2 Inhibitor, in Primary Dysmenorrhea: A Randomized Controlled Trial. Obstet. Gynecol..

[48] Roumie C.L., Mitchel E.F., Kaltenbach L., Arbogast P.G., Gideon P., Griffin M.R. (2008). Nonaspirin NSAIDs, Cyclooxygenase 2 Inhibitors, and the Risk for Stroke. Stroke.

[49] Inanmiş R.A., Deveci S., Yardim T. (2006). Exploration of the Efficacy of Placebo, Lornoxicam and Alverine Citrate+simethicone in the Treatment of Primary Dysmenorrhea. Jinekoloji Obstet. Derg..

[50] Dębska M., Mazurek M., Niemiec T., Dębski R. (2007). Comparative Efficacy and Tolerability of Drotaverine 80 mg and Ibuprofen 400 mg in Patients with Primary Dysmenorrhoea–Protocol DOROTA. Ginekol. Pol..

[51] Weiser T., Richter E., Hegewisch A., Muse D.D., Lange R. (2018). Efficacy and Safety of a Fixed-Dose Combination of Ibuprofen and Caffeine in the Management of Moderate to Severe Dental Pain after Third Molar Extraction. Eur. J. Pain.

[52] Derry S., Wiffen P.J., Moore R.A. (2015). Single Dose Oral Ibuprofen plus Caffeine for Acute Post-operative Pain in Adults. Cochrane Database Syst. Rev..

[53] Kleczar K., Cieplucha A. (2019). A Pharmaceutical Composition Comprising Metamizole, Drotaverine, and Caffeine. European Patent.

[54] Madhu C., Mahavarkar S., Bhave S. (2010). A Randomised Controlled Study Comparing Drotaverine Hydrochloride and Valethamate Bromide in the Augmentation of Labour. Arch. Gynecol. Obstet..

[55] Ibrahim M.I., Alzeeniny H.A., Ellaithy M.I., Salama A.H., Abdellatif M.A. (2014). Drotaverine to Improve Progression of Labor among Nulliparous Women. Int. J. Gynecol. Obstet..

[56] Al-Assadi A. (2015). The Use Of Diclofenac For Pain Relief in the First Stage of Labour. https://www.researchgate.net/publication/311543458_THE_USE_OF_DICLOFENAC_FOR_PAIN_RELIEF_IN_THE_FIRST_STAGE_OF_LABOUR.

[57] Garg U., Chauhan S., Nagaich U., Jain N. (2019). Current Advances in Chitosan Nanoparticles Based Drug Delivery and Targeting. Adv. Pharm. Bull..

[58] Armenta M.E.G., Murillo J.S., Ochoa V.G.Á. (2009). Composición Farmacéutica Compuesta Por La Combinación de Diversos Agentes Antiespasmódicos y Un Agente Antiinflamatorio No Esteroideo, Útil Para El Control y Tratamiento Del Cólico Renal y La Inflamación.

